# Fucoidan alleviated autoimmune diabetes in NOD mice by regulating pancreatic autophagy through the AMPK/mTOR1/TFEB pathway

**DOI:** 10.22038/IJBMS.2023.68739.14981

**Published:** 2024

**Authors:** Haiqi Gao, Yifan Zhou, Chundong Yu, Guifa Wang, Wenwei Song, Zixu Zhang, Lu Lu, Meilan Xue, Hui Liang

**Affiliations:** 1 Department of Biochemistry and Molecular Biology, School of Basic Medicine, Qingdao University, 308 Ningxia Road, Qingdao 266071, PR China; 2 Qingdao No.17 Middle School, 80 Hangzhou Road, Qingdao 266031, Shandong Province, PR China; 3 Department of Laboratory, Women and Children’s Hospital of Qingdao, Qingdao, Shandong 266034, PR China; 4 Department of Human Nutrition, College of Public Health, Qingdao University, Qingdao 266071, PR China; # These authors contributed eqully to this work

**Keywords:** Apoptosis, Autophagy, Fucoidan, NOD mice, Type 1 diabetes

## Abstract

**Objective(s):**

The present study investigated the effect and its underlying mechanisms of fucoidan on Type 1 diabetes mellitus (T1DM) in non-obese diabetic (NOD) mice.

**Materials and Methods:**

Twenty 7-week-old NOD mice were used in this study, and randomly divided into two groups (10 mice in each group): the control group and the fucoidan treatment group (600 mg/kg. body weight). The weight gain, glucose tolerance, and fasting blood glucose level in NOD mice were detected to assess the development of diabetes. The intervention lasted for 5 weeks. The proportions of Th1/Th2 cells from spleen tissues were tested to determine the anti-inflammatory effect of fucoidan. Western blot was performed to investigate the expression levels of apoptotic markers and autophagic markers. Apoptotic cell staining was visualized through TdT-mediated dUTP nick-end labeling (TUNEL).

**Results:**

The results suggested that fucoidan ameliorated T1DM, as evidenced by increased body weight and improved glycemic control of NOD mice. Fucoidan down-regulated the Th1/Th2 cells ratio and decreased Th1 type pro-inflammatory cytokines’ level. Fucoidan enhanced the mitochondrial autophagy level of pancreatic cells and increased the expressions of Beclin-1 and LC3B II/LC3B I. The expression of p-AMPK was up-regulated and p-mTOR1 was inhibited, which promoted the nucleation of transcription factor EB (TFEB), leading to autophagy. Moreover, fucoidan induced apoptosis of pancreatic tissue cells. The levels of cleaved caspase-9, cleaved caspase-3, and Bax were up-regulated after fucoidan treatment.

**Conclusion:**

Fucoidan could maintain pancreatic homeostasis and restore immune disorder through enhancing autophagy via the AMPK/mTOR1/TFEB pathway in pancreatic cells.

## Introduction

Type 1 diabetes mellitus (T1DM) is a preventable metabolic disorder, which is also an epidemic health issue worldwide. The upward trend of persons suffering from T1DM, specifically children and adolescents, is a major worldwide public health concern that calls for appropriate preventive interventions (1). Many studies have found that inflammation may play a crucial role in autoimmune destruction of beta cells of the islets (2, 3). Over the past 20 years, research has identified several immune cell types to destroy insulin-producing β cells (4). T1DM is typically regarded as a progressive autoimmune disorder characterized by T cell mediated islet β cell dysfunction or death (5, 6). The immune imbalance caused by the suppression of Th2 cells and the over-activation of Th1 cells has been proposed as a critical contributing factor in the pathogenesis of T1DM (7). Th1 subgroup and its cytokines interferon-*γ* (IFN-*γ*), tumor necrosis factor-α (TNF-α), and interleukin-1β (IL-1β) may trigger a cascade of inflammatory processes and activate cytotoxic T cells to produce excessive reactive oxygen species (ROS), resulting in disturbed β cell function and impaired insulin secretion (7-9). The serum levels of TNF-α and IFN-γ in patients with T1DM were increased. *In vivo* studies demonstrated that the combination therapy of anti-TCR with anti-IL-1β and anti-TNF-α could alleviate islet inflammatory injury and regain normal glucose levels (10, 11). Therefore, it has been demonstrated that correcting the imbalance of Th1/Th2 is a reliable way to prevent pancreatic islet inflammation in T1DM and treat autoimmune diabetes. 

One promising biomaterial, fucoidan, is a bioactive sulfated polysaccharide extracted from brown seaweeds with a variety of activities, such as anti-oxidative, anticancer, and antiinflammatory (12-14). Cheng *et al*. confirmed that in streptozotocin (STZ)-induced diabetic rats fucoidan could effectively control fasting blood glucose, suppress oxidative stress, and protect against liver injury (15). Several research groups indicated that fucoidan exerted various anti-diabetes effects by inhibiting starch-glucose uptake and restoring lipid homeostasis (19). Furthermore, fucoidan ameliorated diabetes-induced liver and renal damage by decreasing proinflammatory cytokine production and oxidative stress (20). We previously reported that fucoidan could reduce inflammatory damage to islet cells in non-obese diabetic (NOD) mice by regulating intestinal microecology (21). However, the effects of fucoidan on the intracellular environment of the pancreas and autophagy level on maintaining homeostasis under diabetic conditions are still unclear. 

Exposure of islet cells to a high glucose environment makes them susceptible to mitochondrial stress and dysfunction (22, 23). Emerging evidence showed that accumulation of damaged mitochondria due to deficient autophagy or apoptosis could provoke further amplification of inflammation and metabolic disorder in T1DM (24-26). Though the loss of β cells is traditionally regarded as the main cause of insulin deficiency, it is reported that β cells stress is the trigger of an autoimmune response, and apoptosis of disturbed β cells may be an effective pathway to relieve islet injury (27). Furthermore, autophagy may help β cells cope with environmental pressure and return to cellular homeostasis (28-30). There was evidence suggesting that AMP-activated serine/threonine protein kinase (AMPK) negatively regulates the mammalian target of rapamycin (mTOR) to promote autophagy, which is crucial in preventing T1DM (31, 32). Activation of AMPK and autophagy may regulate chronic inflammation and improve the islet environment in diabetic C57BL/6 mice (24) (33). In the T1DM model induced by STZ, the expression levels of autophagy-related proteins, especially Beclin 1 and microtubule-associated protein 1A/1B -light chain 3 (LC3) were increased, indicating that autophagy had a protective effect on islet cells in the early stage of T1DM (34). Recently it has been found that fucoidan may regulate the processes of autophagy (35). In foam cells, fucoidan inhibited lipid accumulation by up-regulating autophagy through increasing the expression of transcription factor EB (TFEB) (36). Our previous study also found that in breast cancer cells fucoidan induced autophagy via regulating the m-TOR/TFEB pathway (37). However, whether fucoidan has a regulatory effect on autophagy in diabetic pancreatic cells remains unclear.

Therefore, we aimed to further explore the protective role and its possible mechanisms of fucoidan on pancreatic cells in spontaneous diabetes. We hypothesized that fucoidan may exert a favorable effect to ameliorate T1DM by inhibiting autoimmune destruction and promoting autophagy of pancreatic β cells to modify the pancreatic microenvironment. In the present study, we intervened NOD mice with fucoidan and then observed its effect on blood glucose and inflammatory factors levels, as well as autophagy and apoptosis-related proteins to further investigate the protective role of fucoidan in the pathogenesis of T1DM.

## Materials and Methods

***Animals and experimental design***

The experiment was approved by the Experimental Animal Care and Use Committee of Qingdao University of Medicine and complied with the Guide for the Care and Use of Laboratory Animals (NIH publication, 8th edition, 2011). Twenty specific pathogen-free female NOD mice (6 weeks old, 12–15 g) were obtained from the Shanghai Laboratory Animal Center, Chinese Academy of Sciences. All mice were housed under controlled temperature (22±3 °C) and humidity of 60% in the 12 hr light and dark cycle. All animals were given free access to water and the same batch of standard laboratory diet.

After one week, the animals were randomly divided into two groups (ten mice in each group): the control group and the fucoidan treatment group. The NOD mice in the control group were administrated normal saline intraglastrically daily, and the fucoidan treatment group was given 600 mg/kg body weight fucoidan (Sigma-Aldrich St. Louis, MO, USA) via i.g. once a day. Body weight was recorded each week. The blood was taken from the tail vein each week to assess the development of diabetes. The intervention lasted for 5 weeks. At last, the 12-week-old mice in each group were subjected to an intraperitoneal glucose tolerance test (IPGTT) and then sacrificed. Spleen and pancreas were collected for subsequent analysis.

***PGTT***

All NOD mice per group were fasted overnight (8 hr), weighed, and administered 1 g/kg glucose solution intraperitoneally. Blood samples were collected from the tail vein at 0, 0.5 , 1 , 2 , and 3 hr after the glucose load. Blood glucose concentration was detected by a glucometer (Accu-Chek, Switzerland).

***Histopathological observation of pancreas***

*Hematoxylin-eosin staining*

Pancreas tissues were taken and fixed in 4% paraformaldehyde. After 24 hr, the tissues were embedded in paraffin and then cut into sections (5 μm thick) by a rotary microtome (Leica RM 2135, Wetzlar, Germany). Hematoxylin-Eosin was used to stain all sections. Finally, sections were observed under a light microscope (Olympus BX50, Tokyo, Japan).

*Transmission electron microscopy*

Pancreatic tissue was collected and fixed at 4 °C for 24 hr with 2.5% glutaraldehyde. After three washes with phosphate buffer saline (PBS), the tissue was fixed with 1% osmium tetroxide for 80 min, dehydrated with standard series acetone concentrations, and then embedded in epoxy resin (SPI Chem/-SPI-PON 812 KIT, West Chester, PA, USA). Semi-thin slices of tissue were obtained by 1% toluidine blue treatment for evaluation and localization. Ultra-thin slices were then cut with 3% uranyl acetate and lead citrate. Finally, the autophagy body in each group was observed under a transmission electron microscope.

***Flow cytometry***

The spleen tissues were placed in a culture dish containing 5 ml of RPMI-1640 medium (HyClone, Logan, UT, USA). A sterile needle core was used to gently ground the tissues. After 100-mesh nylon mesh filtration, cell suspensions were collected. PBS was used to wash the cells three times. Then, the cell concentration was adjusted to 10^6^/ml. Spleen single-cell suspension was initially stained with anti-CD4-FITC and permeabilized with Cytofix/Cytoperm, followed by staining with anti-IL-4-PE and anti-IFN-γ-PE. The antibodies were obtained from Merck-Millipore (Darmstadt, Germany). Finally, the cells were incubated in darkness at 4 °C for 30 min. Flow cytometry was used to measure the ratio of Th1 to Th2 cells

***ELISA***

The levels of IL-6, IL-1β, TNF-α, and IFN-γ in the spleen were quantified by ELISA kits (Cloud-Clone Corp, Houston, USA) according to the manufacturer’s instructions. Spleen tissue was treated in Tris-HCl buffer containing protease inhibitor to prepare into homogenates. After centrifuging at 4 °C for 10 min at 9168 g, the supernatant was taken. Cytokine levels in the supernatant of tissue samples were determined by a specific ELISA kit (Cloud-Clone, USA) according to the manufacturer’s instructions. 

***TUNEL assay***

Apoptosis cells in islet tissues were determined using the TUNEL assay. Briefly, the paraffin-embedded pancreas was gradient hydrated with ethanol, then permeated with protease K (20 μg/ml) at room temperature for 20 min. After washing with Tris-buffered saline (TBS), the sections were incubated at room temperature with TdT balancing buffer for 20 min and then with TdT labeled reaction mixture for 1.5 hr at 37 °C. After washing with TBS, sections were treated with a fluorescein-Fragel™ adherent medium. For counterstaining, the sections were incubated with DAPI for 15 min. Finally, after washing with PBS, the sections were sealed with an anti-fluorescence quenching sealing solution. The sections were observed using a fluorescent microscope for imaging, and TUNEL-positive cells were visualized by green fluorescence.

***Western blotting***

Proteins were extracted from pancreatic tissues with a Protein Extraction Kit (Beyotime Institute of Biotechnology, Jiangsu, China). Also, protein concentration was measured by a bicinchoninic acid (BCA) protein assay kit (Biorbyt, Cambridge, UK). The samples were resolved by sodium dodecyl sulfate-polyacrylamide gel electrophoresis (SDS-PAGE) and electrophoretically transferred to a polyvinylidene difluoride (PVDF) membrane (Seebio, Shanghai, China). After blocking with 5% non-fat milk powder in TBST solution for 1 hr, membranes were incubated with primary antibodies: anti-LC3B, phospho(p)-AMPK, p-mTOR1, TFEB, Beclin1, Bax, Bcl-2, caspase-9, cleaved-caspase-9, caspase-3, and cleaved-caspase-3 at 4 °C overnight. Followed by washing with TBST twice, the corresponding secondary antibody was added and incubated for 1 hr at 37 °C. Meanwhile, the anti-β-actin antibody was used as an internal control. The analysis of protein blots was done with the Image J software.

***Immunofluorescence***

Paraffin-embedded pancreatic tissue sections were deparaffinized and hydrated by a graded series of ethanol followed by repair under high pressure for 5 min, and then slowly cooled to room temperature. Subsequently, sections were incubated with 3% hydrogen peroxide for 20 min. Then the slides were incubated with anti-TFEB antibody (1:60) at 4 °C overnight after being blocked with 5% bovine serum albumin (BSA) for 30 min. After washing with PBS, the sections were incubated with fluorescent secondary antibodies (1:60) at room temperature in darkness for 30 min. The DAPI dyeing solution (4’,6-diamidino-2-phenylindole, Ybscience, Shanghai, China) was used for staining nuclei at room temperature. After 20 min, the sections were sealed with water-soluble tablet sealing liquid and imaged under a fluorescence microscope (OlympusL, Tokyo, Japan).

***Statistical analysis***

Statistical analyses were performed by Prism 7.0 and SPSS v 23.0. Experimental values were expressed as mean ± SD. Differences between the two groups were compared using a t-test. Mann-Whitney test was used for non-parametric tests. The level of significance was uniformly set at *P*<0.05.

## Results

***Effect of fucoidan on body weight, blood glucose, and glucose tolerance in NOD mice***

**Figure 1:**
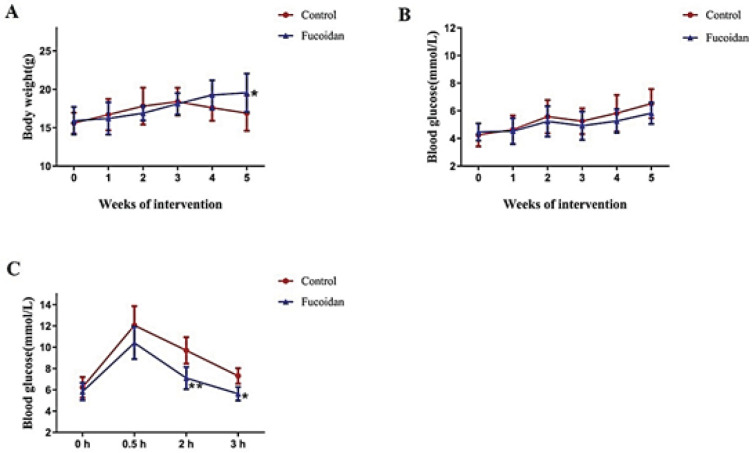
Effect of fucoidan on weight gain, blood glucose levels, and glucose tolerance in NOD mice. (A) Weight gain, (B) Blood glucose level, (C) Glucose tolerance. The data were presented as mean values of n=10 animals/group ± SD. *P<0.05 vs the control group; **P<0.01 vs the control group

In this study, mice’s body weight and blood glucose levels were measured and glucose tolerance was tested to evaluate the effect of fucoidan on T1DM. Treatment with fucoidan markedly improved diabetes symptoms, indicated by gradually increased body weight and decreased blood glucose concentrations (Figure 1). The results showed that the NOD mice in the control group had with body weight gain in the first three weeks and a rapid decline in the following 2 weeks (Figure 1A). The weight gain in the fucoidan treatment group mice increased continuously and was higher than that of control mice in the fifth week (*P*<0.05).

Blood glucose was measured once a week in two groups. The control mice showed progressive increase in blood glucose levels, while in the fucoidan treatment group, the blood glucose level went down following administration of fucoidan (Figure 1B). It suggested that fucoidan could exert hypoglycemic activity in NOD mice. The final level of glucose in fucoidan-treated mice was slightly lower than that in the control group, but there was no statistically significant (*P*>0.05).

As shown in Figure 1C, the mice that received fucoidan had higher blood glucose stability after administering a glucose solution. Compared to control mice, fucoidan treatment significantly lowered blood glucose levels at 2 hr (*P*<0.01) and 3 hr (*P*<0.05) respectively after the glucose load. The results indicated the obvious improvement effect of fucoidan on glucose tolerance.

***Effect of fucoidan on the damage in the pancreas***

**Figure 2:**
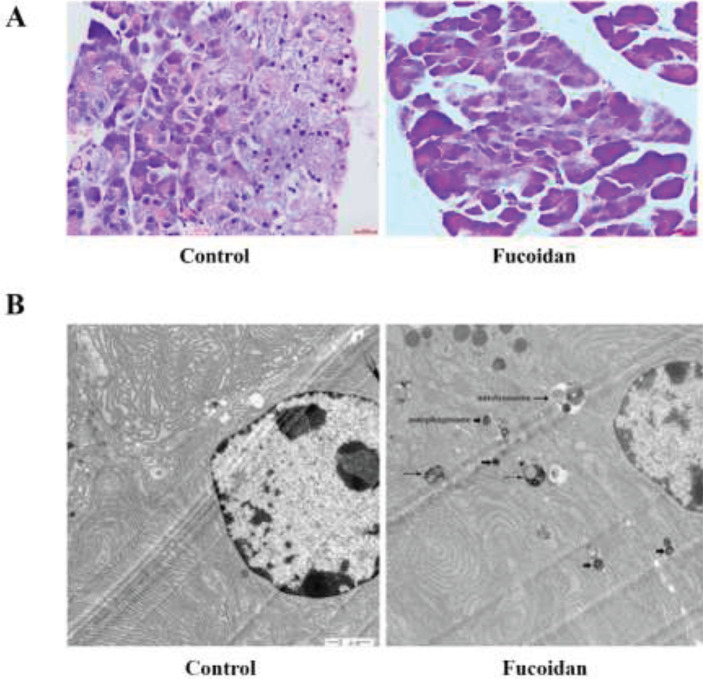
Effect of fucoidan on pancreas structure of non-obese diabetic (NOD) mice

As shown in Figure 2A, spontaneous pancreatic necrosis in NOD mice was alleviated after fucoidan treatment. The control mice showed focal expansion of the interlobular septum, interrupted boundaries, and massive infiltration of inflammatory cells in the pancreas tissue examined by hematoxylin and eosin. Islets of NOD mice treated with fucoidan were distributed evenly with clear boundaries. TEM results showed increased autophagosome with double film and autolysosome of β cells in NOD mice after fucoidan intervention (Figure 2B). 

***Effect of fucoidan on the proportion of Th1 and Th2 cells***

**Figure 3:**
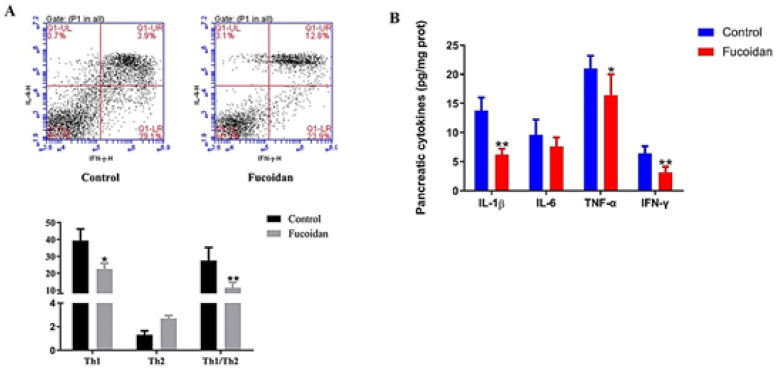
Levels o f inflammation factors of non-obese diabetic (NOD) mice

Th1 cells, the CD4+T cell subgroup, mainly secrete cytokine IFN-γ, while Th2 cells mainly secrete IL-4. The proportion of IFN-γ positive and IL-4 positive spleen T cells was detected by flow cytometry to examine the effect of fucoidan on the proportion of Th1 and Th2 cells. Here, we found that fucoidan intervention could significantly increase the production of IFN-γ and inhibit the level of IL-4 and then notably down-regulated the IFN-γ/IL-4 ratio (*P*<0.05) (Figure 3A). Furthermore, a notable decrease was observed in Th1 type cytokines including IL-1β, IFN-γ, and TNF αderived from pancreas tissue in the fucoidan group, compared to control mice (*P*<0.05, Figure 3B). The data suggested that fucoidan caused a significant bias toward Th2 cell responses and somewhat restored the immune disorder in NOD mice.

***Fucoidan inhibited apoptosis of pancreatic β cells***

**Figure 4:**
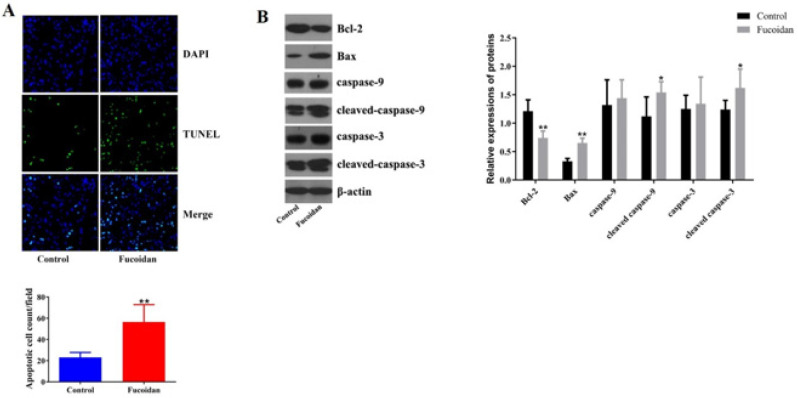
Effect of fucoidan on pancreatic cell apoptosis of mice

TUNEL staining was performed in pancreatic tissues to evaluate the effect of fucoidan on pancreatic β cell apoptosis. The pancreatic tissues in the fucoidan group showed stronger TUNEL-positive-cell-staining compared to the control group observed under the fluorescence microscope. We counted an elevation of 2.6 folds in apoptotic islet cells number in pancreatic tissues of the fucoidan group compared to control mice (Figure 4A). Furthermore, western blot analysis revealed that Bcl-2 decreased after fucoidan treatment, together with an increase in Bax protein. Caspase-9 and caspase-3, which are key molecules in the apoptotic pathway, were highly activated after fucoidan treatment (Figure 4B). Our data showed that fucoidan could induce apoptosis in pancreatic β cells.

***Effect of fucoidan on autophagy of pancreatic cells in NOD mice***

**Figure 5:**
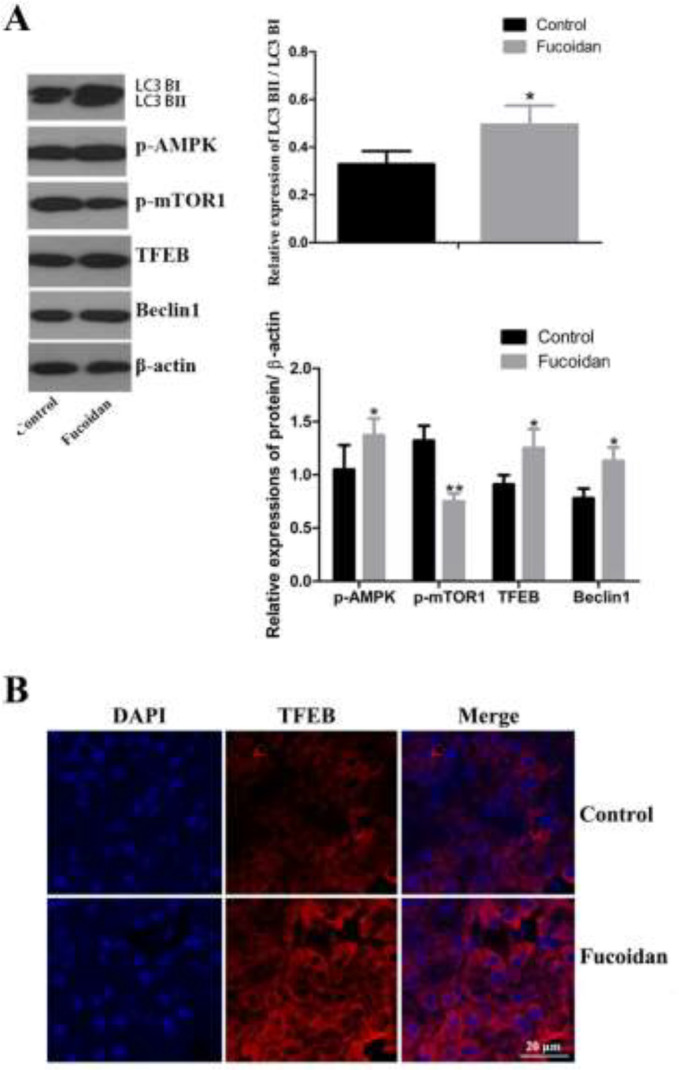
Effect of fucoidan on molecules of autophagy pathway in mice

Fucoidan intervention remarkably up-regulated the total abundance of LC3 B and the ratio of LC3B II/LC3B I (*P*<0.05). Increased protein contents of p-AMPK, Beclin 1, and TFEB were observed in fucoidan-treated mice compared to control mice, while p-mTOR1 expression was down-regulated (Figure 5A). TFEB is a transcription factor to positively regulate target genes involved in autophagy. We further confirmed up-regulation of nuclear localization of TFEB in β cells by immunofluorescence assay (Figure 5B). Change in these marker proteins and autophagosome suggested that fucoidan-induced autophagy activity and AMPK/mTOR1/TFEB pathway may involve autophagy regulation of β cells.

## Discussion

Disorders in the immune system and dysfunction in β cells of the pancreas are key contributors to the pathogenesis of T1DM (2). It has been reported that apoptosis and autophagy processes can effectively improve β cells’ vitality and protect islet cells from inflammatory assaults (27, 28). In the present work, the data showed that after 5 weeks of fucoidan treatment the glucose tolerance was improved and blood glucose was decreased in NOD mice. The results of HE staining of the pancreas displayed that fucoidan improved the pancreatic tissue structure and inhibited necrosis of pancreatic cells. Fucoidan corrected the imbalance of Th1/Th2 through down-regulating of Th1 proinflammatory cytokines levels and inducing of Th2-biased cytokine response. Additionally, fucoidan improved β cells’ vitality via enhancing mitochondrial autophagy and apoptosis, which may suppress inflammation and maintain the internal environment of the pancreas.

During T1DM development, pancreas injury is proven to be mainly related to aberrant inflammation mediated by abnormal Th1/Th2 ratios (5, 6). As our previous study, fucoidan suppressed Th1 type immune response in NOD mice via inducing the generation of CD4 + CD25+ Foxp3+ Tregs, suggesting the immune balance of Th1/Th2 cells induced by fucoidan (21). Immunomodulatory effects of fucoidan have been studied in various autoimmune diseases. These studies showed that fucoidan as an exogenous sulfated polysaccharide, could regulate inflammation and improve inflammatory events (38). Moreover, fucoidan could inhibit the production of autoreactive T cell response and inflammatory cytokine TNF-α, thus improving the clinical paralysis in experimental autoimmune encephalomyelitis rats. A study by Zunhua Shu showed that fucoidan inhibited survival and induced apoptosis in IL-1β-treated rheumatoid arthritis fibroblast synoviocytes (39). Similarly, we further confirmed the immunomodulatory effects of fucoidan in autoimmune diabetes. 

The spleen cytokines IL-4 and IFN-γ were determined by flow cytometry to further confirm the regulating effect of fucoidan on the proportions of Th1/Th2 cells. Results of the current study revealed that fucoidan intervention significantly increased the ratio of spleen IL-4/IFN-γ in T1DM mice and thus partly restored the Th1/Th2 balance. Moreover, the levels of Th1 inflammatory mediators including IL-6, IL-1β, IFN-γ, and TNF-α decreased in pancreatic tissue after fucoidan intervention, indicating that fucoidan could relieve Th1 cells and Th1 cytokine-mediated pancreatitis locally. These results were consistent with previously published studies. More recently, one antigen-specific therapy utilized GAD65 in combination with aluminum hydroxide and took advantage of alum’s Th2-biased adjuvant property, thereby modulating pathological Th1 autoimmunity by redirecting the dominant Th1 response to the Th2 response (40). Promoting Th1 cell/cytokine-based immune response transforms into a dominant Th2 cell/cytokine response by fucoidan is expected to be a promising strategy to inhibit the development of T1DM.

However, the pattern of Th1/Th2 imbalance is insufficient to explain the immunopathology of autoimmune diabetes. The dysfunction and even death of β cells are key contributors to the disease (41). Stressed β cells secrete chemokine, which may play a major role in islet inflammation. Enhanced apoptosis of disturbed β cells is seen as a protection mechanism to inhibit the amplification of inflammation. Extensive studies demonstrated that fucoidan could induce mitochondrial apoptosis in a number of cell lines by up-regulating the Bax/Bcl-2 ratio and inducing cleavage of caspase-9 and caspase-3 (42) (43). Similarly, we found that fucoidan may trigger cell apoptosis through a caspase-dependent pathway by up-regulating Bax and down-regulating Bcl-2. In addition, fucoidan-induced apoptosis involved the up-regulation of AMPK phosphorylation, which promoted the activation of caspase 9 and caspase 3. 

Up-regulation of AMPK has also been viewed as a central role in cell autophagy (44). AMPK induced autophagy by inhibiting mTOR1, promoted dephosphorylation of the TFEB into the nucleus, and stimulated the expression of the downstream autophagy-related gene Beclin1 to mediate autophagy, and it could also promote lipid efflux and inhibit the release of inflammatory factors. Indeed, autophagy was involved in restoring cellular homeostasis under environmental pressure (45). Regulation of β cell quality and function by elective phagocytosis of damaged mitochondria and other impaired organelles via autophagosomes is of paramount importance in promoting β cell health and preventing the progression of diabetes (46). Oxidative stress and increased glycemic load induced insufficient autophagy and enhanced the aggregation of dysfunctional mitochondria, thus resulting in disrupted glucose or lipid metabolism and acceleration of T1DM disease progression (47). We assessed the phosphorylation levels of mTOR1 and AMPK in pancreatic β cells of NOD mice. This study revealed that fucoidan increased the level of p-AMPK and reduced the level of p-mTOR1. Additionally, fucoidan treatment increased LC3B I to LC3B II conversion and induced nuclear translocation of TFEB, indicating that fucoidan activated β cells autophagy in T1DM mice. Our results demonstrated that fucoidan could maintain pancreatic β cell viability by inducing autophagy through the AMPK/mTOR1/TFEB signaling pathway.

Recent advancements in clinical drugs were shown to inhibit the progression of T1DM through modulation autophagy in AMPK/mTOR signaling pathway. Liraglutide enhanced autophagy by activating AMPK/mTOR pathway and reduced cognitive impairment in STZ-induced diabetic mice (48). Activation of autophagy via the AMPK/mTOR pathway could alleviate the cognitive impairment caused by conventional protein kinase C (cPKC)γ deficiency in T1DM mice (49). Guo *et al*. reported that acetaldehyde dehydrogenase-2 (ALDH2) played a protective role against diabetes-induced cardiotoxicity and myocardial dysfunction by activating the AMPK-dependent autophagy process (50). The above studies have confirmed that autophagy induction via AMPK/mTOR pathway acts as a promising strategy to rescue pancreatic β cell dysfunction and relieve diabetes complications. In addition, other studies reported that inhibiting the mTORC1 activity of CD4+T cells with rapamycin may decrease the differentiation of Th1 and Th17 cells and activate Th2 cell differentiation (51). Overall, we suggested that fucoidan alleviated inflammation via promoting apoptosis and autophagy targeting dysfunctional β cells, in combination with inhibiting islet autoimmunity, which proposed a feasible therapy for preventing T1DM.

NOD: Non-obese diabetic

(A) H&E staining (B) Electron microscope observation. The arrows indicated autolysosome and autophagosome in the pancreas.

(A) Flowcytometric analyses (B) Levels of Th1 type cytokines, IL-1β, IFN-γ, and TNF-α in the spleen of NOD mice.

*P<0.05 vs the control group; **P<0.01 vs the control group

(A) Effects of fucoidan on the levels of LC3 B, p-mTOR1, p-AMPK, Beclin 1, and TFEB in pancreatic cells, (B) Immunofluorescence assay of TFEB.

*P<0.05 vs the control group; **P<0.01 vs the control group

## Conclusion

As shown in this study, fucoidan reduced blood glucose levels and modified the pancreatic microenvironment mainly via improving the disorder of Th1/Th2 and enhancing autophagy and apoptosis of pancreatic β cells by the AMPK/mTOR1/TFEB signaling pathway. It was suggested that fucoidan may become a supplementary agent in preventing T1DM. However, apoptosis and autophagy are controlled and regulated by complex signaling pathways *in vivo*, the effect of fucoidan on β cells cannot be clarified only through animal models alone. So further examination is needed to explore the protective mechanism of fucoidan on T1DM.

## Authors’ Contributions

M X and H L contributed to the conception and design of the research; H G, Yifan ZhoY Zu, and Z Z performed the experiments; C Y and G W analyzed the data and interpreted the results of the experiments; Y Z, W S, and L L prepared the figures and drafted the manuscript; M X and H L revised the final version of the manuscript.

## Conflicts of Interest

The authors declare that they have no competing interests.
